# Anthracycline-containing chemotherapy causes long-term impairment of mitochondrial respiration and increased reactive oxygen species release in skeletal muscle

**DOI:** 10.1038/srep08717

**Published:** 2015-03-03

**Authors:** Gilles Gouspillou, Celena Scheede-Bergdahl, Sally Spendiff, Madhusudanarao Vuda, Brian Meehan, Heather Mlynarski, Elodie Archer-Lahlou, Nicolas Sgarioto, Fennigje M. Purves-Smith, Yana Konokhova, Janusz Rak, Stéphanie Chevalier, Tanja Taivassalo, Russell T. Hepple, R. Thomas Jagoe

**Affiliations:** 1 McGill University Health Centre, Montreal, Quebec, Canada; 2 Département de Kinanthropologie, Université du Québec à Montréal, Montreal, Quebec, Canada; 3 Lady Davis Institute for Medical Research, Jewish General Hospital, Montreal, Quebec, Canada; 4 Department of Kinesiology, McGill University, Montreal, Quebec, Canada; 5 Department of Oncology, McGill University, Montreal, Quebec, Canada; 6 Montreal Children's Hospital Research Institute, Montreal, Quebec, Canada

## Abstract

Anticancer treatments for childhood acute lymphoblastic leukaemia (ALL) are highly effective but are now implicated in causing impaired muscle function in long-term survivors. However, no comprehensive assessment of skeletal muscle mitochondrial functions in long-term survivors has been performed and the presence of persistent chemotherapy-induced skeletal muscle mitochondrial dysfunction remains a strong possibility. Non-tumour-bearing mice were treated with two drugs that have been used frequently in ALL treatment (doxorubicin and dexamethasone) for up to 4 cycles at 3-week intervals and euthanized 3 months after the 4th cycle. Treated animals had impaired growth and lower muscle mass as well as reduced mitochondrial respiration and increased reactive oxygen species production per unit oxygen consumption. Mitochondrial DNA content and protein levels of key mitochondrial membrane proteins and markers of mitochondrial biogenesis were unchanged, but protein levels of Parkin were reduced. This suggests a novel pattern of chemotherapy-induced mitochondrial dysfunction in skeletal muscle that persists because of an acquired defect in mitophagy signaling. The results could explain the observed functional impairments in adult survivors of childhood ALL and may also be relevant to long-term survivors of other cancers treated with similar regimes.

Despite the successes of modern treatments for childhood acute lymphoblastic leukaemia (ALL)^1^,^2^, the poor long-term health of survivors of childhood cancers, including ALL, is of increasing concern^3^. Many survivors of childhood cancer have impaired functional capacity and a substantially increased risk of chronic disease and early mortality^3^. The chemotherapy agents used as part of the anti-ALL treatment are implicated as one of the likely causes for these long-term effects in skeletal muscle, including reduced muscle mass and function (reviewed in Ref. 4). However, the mechanism(s) of longer term chemotherapy-induced reduction in skeletal muscle mass, strength and endurance in survivors of ALL is unclear.

The anthracyclines are still commonly used for the treatment of ALL^5^ as well as in other malignancies. The use of these drugs has been restricted more recently, especially for low risk disease, as they are now known to cause cardiac muscle damage which can become evident several years after treatment has finished^6^. In rodents, repeated dosing with doxorubicin leads to progressive accumulation and substantially higher drug levels in both cardiac and skeletal muscle, compared with other tissues such as the liver^7^. The preferential accumulation of anthracyclines in muscle tissues is thought to be due to very strong binding to proteins such as cardiolipin in the inner mitochondrial membrane and iron-containing protein complexes, both of which are abundant in muscle tissues^8^. Long-term, persistent anthracycline-induced cardiac muscle mitochondrial dysfunction is mediated by oxidative stress-induced mitochondrial DNA (mtDNA) deletions^9^,^10^,^11^,^12^. In rats, accumulation of mtDNA deletions impairs transcription and expression of mtDNA-encoded components of the electron-transport chain, such as cardiac muscle mtDNA-encoded cytochrome c oxidase (COX), subunit I^11^. Similarly in a small human autopsy study, doxorubicin-treated patients had reduced mtDNA to nuclear DNA ratio, increased rates of the common mtDNA deletion and reduced COX to succinate dehydrogenase (SDH) activity ratio in cardiac muscle compared to controls^13^.

In contrast, prior studies have concluded that skeletal muscle is resistant to the longterm damaging effects of anthracyclines. For example, the cardiac muscle markers of reduction in mitochondrial function and increased levels of mtDNA deletions^11^,^13^ were not replicated in skeletal muscle. In fact Lebrecht et al^11^ reported unchanged levels of COXI protein in skeletal muscle in rat skeletal muscle 30 weeks after completion of anthracycline treatment. Similarly, skeletal muscle does not demonstrate the same transcriptional suppression of sarcomeric gene expression as cardiac muscle after anthracycline treatment^14^. However, one study reported an accelerated decline in complex I activity in diaphragm muscle several weeks after completion of doxorubicin treatment in rats, which was similar to that seen in heart muscle from the same animals^15^. Acute treatment of cultured skeletal myotubes^16^ and mice^17^ confirms that the anthracycline, doxorubicin, does cause early increases in skeletal muscle mitochondrial ROS, increased proteolysis^16^ and muscle weakness^17^. It still remains to be determined whether skeletal muscle mitochondria demonstrate any long-term functional impairment after anthracycline treatment, and if so what mechanisms may be involved.

Chemotherapy treatment for ALL is delivered using multi-drug regimes administered in phases using repeating cycles over several months. In addition, other drugs for control or prevention of chemotherapy-induced symptoms, such as nausea and vomiting, are also used. Hence, in seeking to determine whether chemotherapy treatment causes skeletal muscle dysfunction in ALL, the net effect of repeated administration of combinations of different drugs needs to be taken into account. Corticosteroids are widely used as standard anti-emetics with chemotherapy for cancer and, in ALL, high dose corticosteroids have an additional specific antineoplastic role^5^. Prolonged, high-dose corticosteroid use is also well known to cause muscle atrophy and weakness^18^ and mitochondrial dysfunction^19^. However, the long-term sequelae of the repeated, short-term use of corticosteroids alone, or in combination with anthracyclines, has not been studied.

To establish whether the combination of doxorubicin and dexamethasone treatment causes long-term mitochondrial dysfunction in skeletal muscle, we studied non-tumor bearing mice and performed detailed mitochondrial functional testing using saponin-permeabilized skeletal muscle fibers^20^. In addition, we performed assays for muscle mtDNA deletions, mitochondrial structural proteins, proteins involved in regulating mitochondrial biogenesis and degradation, as well as measuring overall muscle size, fiber type and morphology. To enhance the clinical relevance of our results we also incorporated cyclical administration of drugs every 3 weeks for 4 cycles. To study the long-term effects of this combined treatment, the changes in treated and control mice were compared, both during treatment and three months after all treatment had been completed.

## Results

### Repeated doxorubicin and dexamethasone treatment impairs whole body and muscle growth

**Figure 1:**
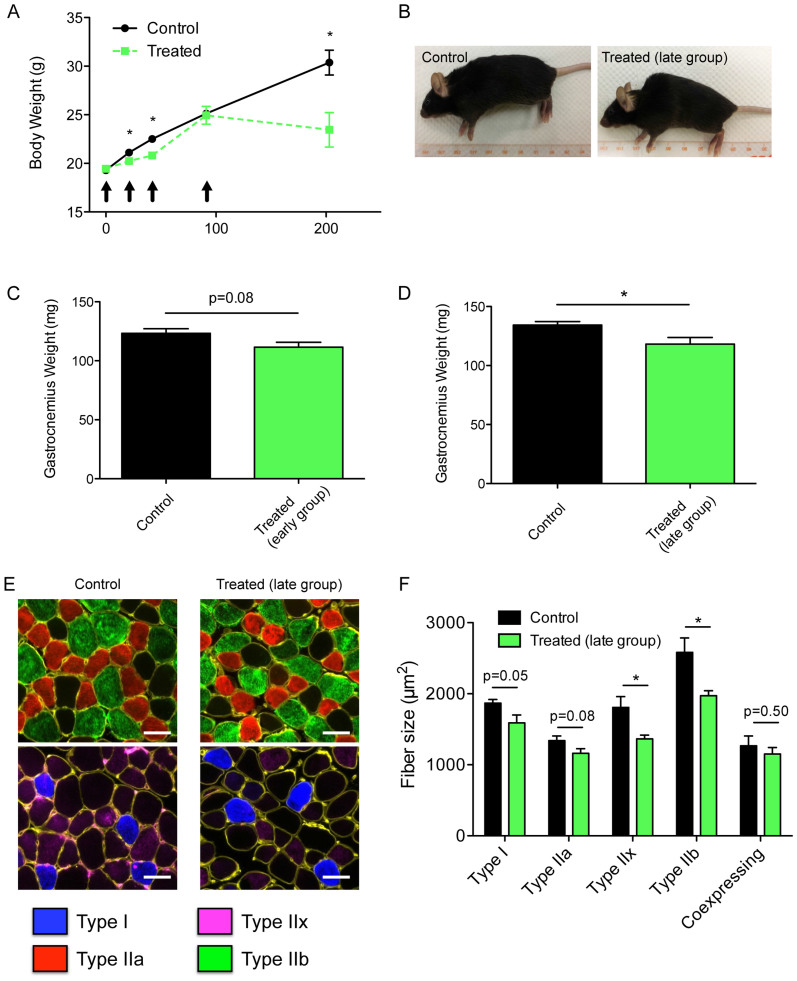
Chemotherapy treatment causes sustained impairment of whole animal and muscle growth. (A) Changes in body weight over time in control vs. chemotherapy treated animals. Arrows indicate timings of each cycle of chemotherapy. (B) Representative photographs of control (left) and treated (right) mice taken at the end of our long-term chemotherapy protocol. Gastrocnemius muscle weights obtained in control and treated animals in the early (C) and late (D) chemotherapy treatment groups. (E) Representative triple MHC labeling (MHCI: blue; MHCIIa: red; MHCIIx: purple; MHCIIb: green) performed on gastrocnemius muscle serial cross sections obtained in control (images on the left) and treated (images on the right) mice in the late group. Scale bars: 50 μm. (F) Quantification of gastrocnemius muscle fiber size in control and treated mice. Data are presented as mean ± SEM. *P < 0.05; **P < 0.01.

Mice treated with repeated cycles of doxorubicin and dexamethasone had impaired growth (Fig 1A). Daily weights and observation immediately after each cycle revealed that treated mice suffered early rapid loss of both weight and physical condition, which recovered to pre-treatment levels over the subsequent two or three weeks following the first two cycles of treatment (data not shown). However, the fourth cycle had to be delayed for some weeks to allow the mice to recover prior to the final treatment cycle. This capacity for compensatory weight gain was lost after the fourth cycle as demonstrated by the plateauing of the growth curve after the last cycle of treatment to the end of the experiment (Fig 1A). Thus, at 3 months after the fourth cycle of chemotherapy treatment, the treated mice weighed 23% less than controls (late group treated vs controls, mean(SD): 23.5(5.6) vs 30.4(4.0) g, P = 0.006) (Fig 1A). Furthermore at sacrifice, many of the chemotherapy-treated mice had patchy hair loss and a prominent dorsal hump, reminiscent of muscular dystrophies and other conditions characterized by skeletal muscle weakness (Fig 1B).

For mice in the early group, euthanized after only two cycles of chemotherapy, the 9% reduction in mean gastrocnemius muscle mass was not statistically significant (P = 0.08, Fig 1C), but at the end of the experiment gastrocnemius muscle mass was 12% lower in the treated group (P = 0.02, Fig 1D). There was no difference in fiber-type proportions (data not shown) but analysis of muscle fiber sizes revealed that treated mice in the late group had fibers which were, on average, 23% smaller (P = 0.009). Although all fibers were somewhat smaller in treated animals, the differences were most marked for the most glycolytic muscle fibers, type IIx and IIb (24% reduction, P = 0.02) (Fig 1E and F).

### Repeated doxorubicin and dexamethasone treatment leads to sustained impairment of maximal mitochondrial respiratory capacity and increased mitochondrial ROS production

**Figure 2:**
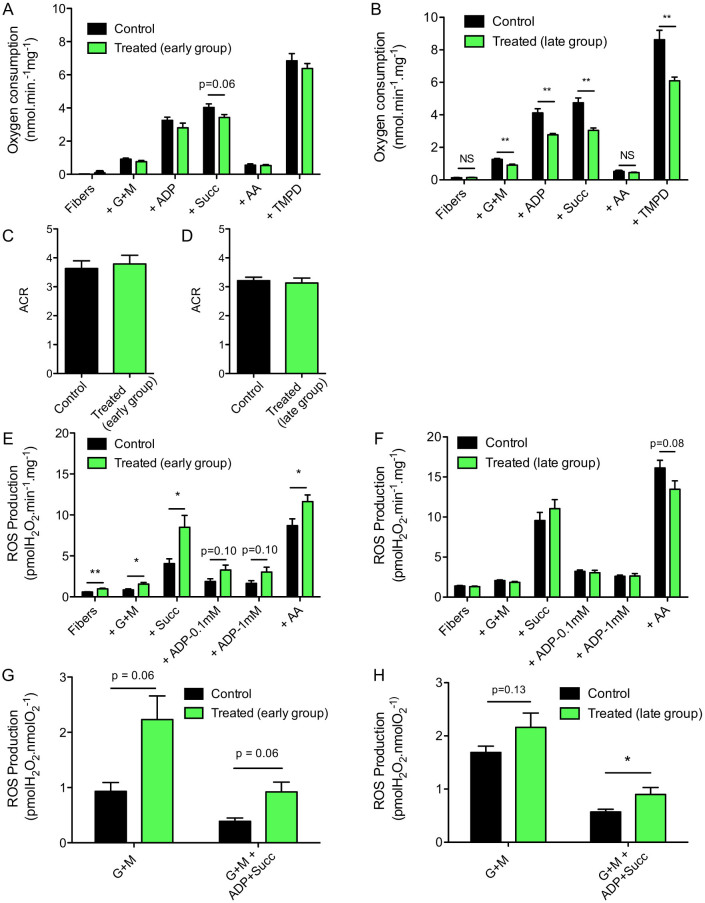
Chemotherapy treatment causes generalized chronic impairment in skeletal muscle mitochondrial function. (A, B) Mitochondrial respiration was assessed in permeabilized myofibers prepared from the red gastrocnemius muscle regions in control and treated mice in early (A) and late (B) groups. Mitochondrial substrates and inhibitors were subsequently added as follows: G + M, ADP, succinate (Succ), AA, and ascorbate + TMPD (TMPD). (C, D) Acceptor control ratio (ACR), a parameter indicative of mitochondrial coupling efficiency, was determined in control and treated mice in early (C) and late (D) groups. ACRs values were obtained by dividing the respiration rate with G + M + ADP (ADP, state 3) by the respiration rate with G + M (state 2). (E, F) Mitochondrial H_2_O_2_ production was assessed in permeabilized myofibers prepared from the red gastrocnemius muscle regions of control and treated mice in early (E) and late (F) groups. Mitochondrial substrates and inhibitors were subsequently added as follows: G + M, succinate (Succ), 0.1 mM ADP, 1 mM ADP, and AA. (G, H) Mitochondrial free radical leak was determined by dividing H_2_O_2_ emission rates by their corresponding respiration rates (data from panel A and B). Data are presented as means ± SEM. *P < 0.05; **P < 0.01.

Mitochondrial respiration was assessed using sequential addition of substrates and inhibitors to distinguish effects on different complexes. Thus basal respiration (state 2) driven by complex I substrates, maximal ADP-stimulated respiration (state 3) driven by complex I (G + M) or complex I + II substrates (G + M + succinate), as well as complex IV respiration (driven by the addition of ascorbate + TMPD following inhibition of complex III using antimycin A) were measured. The early group (euthanized after 2 cycles of chemotherapy) did not have any significant alteration in mitochondrial respiratory function (Fig 2A). In contrast, the respiratory function of mitochondria from animals the late group (euthanized 12 weeks after 4 cycles of chemotherapy) was greatly impaired (Fig 2B). For example, maximal respiratory capacity, driven by complex I and II substrates, was 36% lower (3.0(0.4) vs 4.7 (1.0) nmol/min/mg, P = 0.0001) in treated mice (Fig 2B, Succ). Interestingly, animals from early and late groups displayed similar acceptor control ratio (ACR) values to control animals, indicating that the coupling efficiency of mitochondrial oxidative phosphorylation was not affected by chemotherapy treatment (Fig 2C and D).

The absolute ROS emission per mg muscle tissue under state 2 conditions (basal, ADP-restricted, respiration driven by complex I (G + M) and complex I + II substrates (G + M + Succ)) and the maximal ROS emission, induced by the complex III inhibitor antimycin A (AA), was significantly increased in the early group (Fig 2E). A trend for an increase in state 3 ROS production (during ADP-stimulated respiration with complex I and II substrates) was also observed in these animals (Fig 2E, ADP). In contrast, no difference in absolute ROS production was evident in the late group (Fig 2F). ROS production was then normalized per unit of O_2_ consumption to define the effects of chemotherapy treatment on the fraction of O_2_ molecules lost to free radical leak. As can be seen in Fig 2G, muscle mitochondria from mice from the early group had a strong trend for an increase in free radical leak under both state 2 and 3 conditions (Fig 2G: G + M, G + M + ADP + Succ). However, mice from the late group had a clear (58%) increase in free radical leak under state 3 driven by complex I and II substrates (e.g. mean(SD): 0.90(0.4) vs 0.58(0.16) pmol H_2_O_2_/nmol O_2_, P = 0.03) (Fig 2H, G + M + ADP + Succ) and a trend for an increase in free radical leak under state 2 conditions (Fig 2H, G + M).

Despite these marked differences in respiratory capacity and respiration-associated ROS production found in muscle mitochondria from chemotherapy-treated mice, the calcium retention capacity (CRC) and the time to permeability transition pore (mPTP) opening assessed as described in Ref. 21, were unchanged following long-term chemotherapy treatment (CRC mean(SD): 0.24(0.09) vs 0.27(0.14) nmol Ca^2+^mg^−1^; time to mPTP opening: 130(69) vs 131(62) s, in treated vs control mice, respectively).

### The decreased skeletal muscle mitochondrial respiration observed in chemotherapy-treated mice is not due to reduction in mitochondrial content

**Figure 3:**
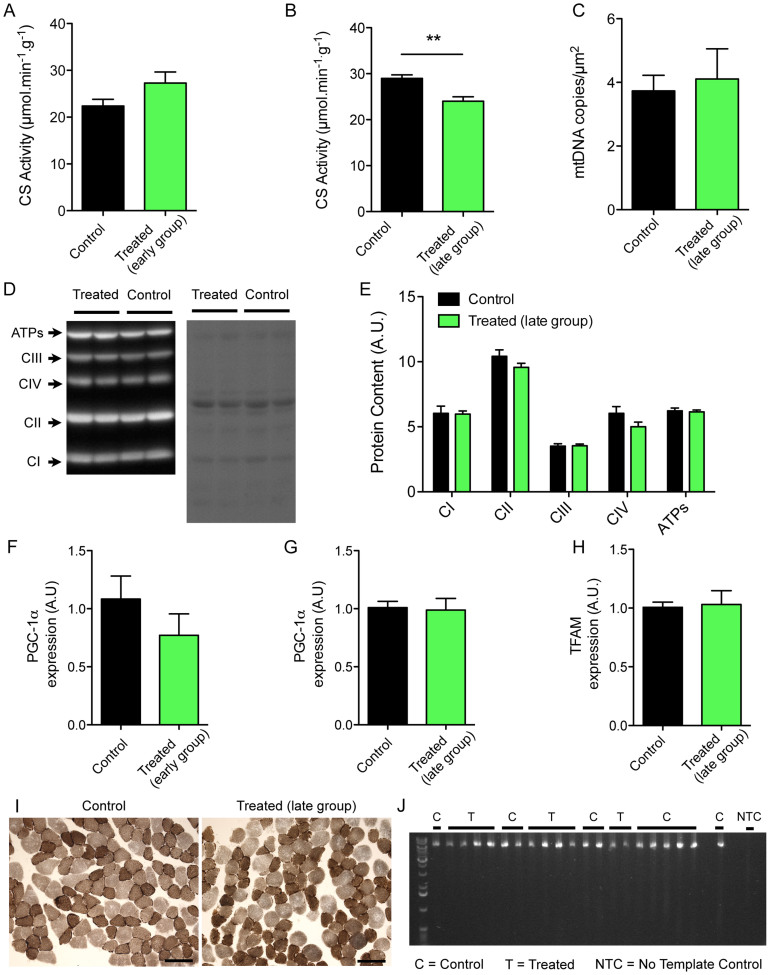
Chemotherapy treatment-induced chronic intrinsic mitochondrial functional changes in skeletal muscle are not mediated by increased mtDNA mutations or reduced mitochondrial content. (A, B) Quantification of citrate synthase activity performed in gastrocnemius muscle of control and treated mice in early (A) and late (B) groups. (C) Quantification of the mitochondrial DNA copy number obtained in the gastrocnemius muscle of control and treated mice in the late group. (D) Left panel: representative immunoblot for complex I (CI), complex II (CII), complex III (CIII), complex IV (CIV) and the ATP synthase (ATPs) performed on gastrocnemius samples obtained from control and treated mice in the late group. Right panel: the corresponding Ponceau stain was used to ensure equal protein loading between lanes. E) Quantification of complex I, II, III, IV and ATPs contents in the gastrocnemius muscles obtained from control and treated mice in the late group. (F, G). Quantification of the relative PGC-1α mRNA expression in the gastrocnemius muscles obtained from control and treated mice in early (F) and late (G) groups. (H) Quantification of the relative PGC-1α mRNA expression in the gastrocnemius muscles obtained from control and treated mice in the late group. (I) Representative sequential complex IV and complex II stain performed in gastrocnemius cross-sections of control (left) and treated (right) mice in the late group. Note that any fibers with high accumulation of mitochondrial DNA mutations should appear blue. Note that out of the 10 treated mice analyzed in the late group, only one showed 2 blue fibers. (J) Long-range PCR amplification of a fragment of the major arc of the mitochondrial DNA, very often affected by mtDNA deletions in pathological conditions, performed on samples from the plantaris of control and treated mice in the late group. Data in graphs are presented as means ± SEM. *P < 0.05.

Citrate synthase activity (CS), a commonly used surrogate for mitochondrial content, was no different between treated and controls in the early group (P = 0.12) and only modestly less than controls in the late group (17% less, P = 0.0008) (Fig 3 A, B) which would suggest a slightly lower mitochondrial content in the late group. However, in contrast, there was no difference in all other markers of mitochondrial content between treated and control mice in the late group. Thus, the mtDNA copy-number in chemotherapy treated mice was similar to control mice (Control vs Chemo, CN/μm^2^ mean (SD): 3.7(1.6) vs 4.4(2.7), P = 0.5) (Fig 3 C), as were the content of inner mitochondrial membrane proteins representing all four complexes of the respiratory chain and the ATP synthase (OXPHOS) (Fig 3D and E). Consistent with this, the mRNA levels of two key transcription factors of the mitochondrial biogenesis program, PGC-1α and TFAM, were unchanged (Fig 3 F, G and H) after chemotherapy treatment. Taken together, our results clearly indicate that neither mitochondrial content nor mitochondrial biogenesis were affected by chemotherapy treatment. Thus, the marked long-term impairment in muscle respiratory capacity and the lower CS activity seen in the late group (Fig. 2B) is not due to a reduction in mitochondrial content and represents a persistent intrinsic mitochondrial respiratory impairment following chemotherapy.

### The impaired skeletal muscle mitochondrial respiration in chemotherapy-treated mice is not caused by mtDNA deletions

Despite evidence of marked global reduction in respiratory capacity in long-term chemotherapy-treated mice there was no evidence of excess accumulation of COX-deficient (blue) fibers in chemotherapy-treated mice (Fig 3I). This suggests mtDNA damage is unlikely to be the cause of impaired mitochondrial respiratory capacity. This observation was corroborated by results of tests for the presence of mtDNA mutations using long-range PCR to amplify the major arc of the mtDNA: the area most often affected by mtDNA deletions in pathological conditions^22^,^23^. The only bands detected corresponded to the wild type form of the mtDNA and there was no evidence to support increased mtDNA deletions (Fig 3J).

### Chemotherapy-induced impairment of mitochondrial function is not associated with increased myocellular oxidative damage but may be exacerbated by reduced mitophagy

**Figure 4:**
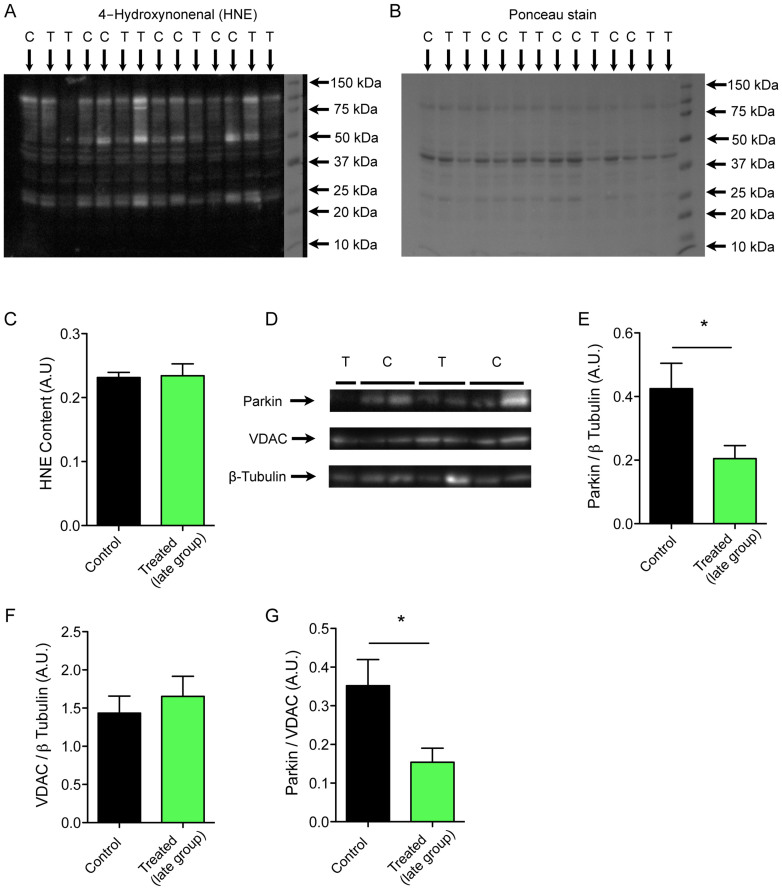
Altered mitochondrial quality control mechanisms rather than increased cellular oxidative damage are implicated in chemotherapy-induced chronic intrinsic mitochondrial dysfunction. (A) Immunoblot for 4-Hydroxynonenal (HNE), a marker of lipid peroxidation used to assess oxidative stress, performed on gastrocnemius muscle samples obtained from control (1) and treated (2) mice in the late group. (B) Corresponding Ponceau stain that was used to ensure equal protein loading between lanes. (C) Quantification of the HNE content in gastrocnemius muscle samples obtained from control and treated mice in the late group. (D) Representative Parkin, VDAC and β-tubulin immunoblots performed on gastrocnemius muscle samples obtained from control and treated mice in the late group. (E, F) Quantification of the protein content of Parkin (E) and VDAC (F), normalized to β-tubulin protein content, in gastrocnemius muscle samples obtained from control and treated mice in the late group. (G) Quantification of the Parkin over VDAC content in gastrocnemius muscle samples obtained from control and treated mice in the late group. Data are presented as means ± SEM. *P < 0.05; **P < 0.01.

Anthracycline-induced cellular damage in cardiac tissue is caused by mitochondrial-derived oxidative stress^24^. Thus we measured the 4-hydroxy-2-nonenal (HNE) content (a stable marker of lipid peroxidation) of whole muscle homogenates. Despite evidence of greatly decreased respiratory capacity and increased respiration-induced free-radical leak, we found no evidence of increased HNE-protein adducts in the late group (Fig 4A, B and C). The ubiquitin ligase, Parkin, is central to mitophagy pathways for the removal of defective mitochondria. Parkin, interacts with the mitochondrial targeted serine-threonine kinase, PINK1^25^ and binds the voltage-dependent anion channel (VDAC) in the outer mitochondrial membrane^26^,^27^, before ubiquitinating VDAC and other proteins on the outer mitochondrial membrane. VDAC levels were unchanged with chemotherapy treatment, but Parkin expression and the ratio of Parkin to VDAC was greatly reduced (51% reduced, P = 0.03 and 53% reduced, P = 0.02 respectively) in late group chemotherapy-treated mice (Fig 4 D-G). This is consistent with a chemotherapy-induced long-term impairment in skeletal muscle mitochondrial quality control pathways.

## Discussion

We have shown that administration of four cycles of doxorubicin and dexamethasone induces a progressive inhibition of growth that becomes irreversible after the fourth cycle of treatment in non-tumor-bearing mice. Three months after the last treatment was given, treated animals demonstrated the typical spinal kyphosis seen in muscle dystrophies and premature aging models^28^,^29^ and had lower muscle mass and a global reduction in muscle fiber cross-sectional area (Fig 1). In light of prior data showing that anthracycline toxicity in cardiac muscle is closely linked to mitochondrial dysfunction, we studied the effects of the dexamethasone and doxorubicin treatment on mitochondrial function in skeletal muscle. Chemotherapy treatment led to a severe impairment in mitochondrial energetics, as shown by the marked reduction in muscle respiratory capacity, and increased mitochondrial ROS production per unit oxygen consumption without any change in mitochondrial sensitivity to permeability transition (Fig 2). The low muscle respiratory capacity observed is not the result of a reduction in mitochondrial content, as there was no reduction in mRNA expression of key transcription factors for mitochondrial biogenesis nor protein components of mitochondrial outer and inner membranes (VDAC and OXPHOS, respectively; Fig 3). Furthermore our studies did not reveal any differences in mtDNA copy number nor evidence of mtDNA deletions in chemotherapy-treated mice. This is in contrast to prior studies in cardiac muscle tissue, where anthracycline toxicity leads to long-term mitochondrial dysfunction by a mechanism that involves increases in mtDNA deletions and reduced mitochondrial volume.

In fact our observations of a marked reduction in skeletal muscle mitochondrial *function* in the absence of corresponding changes in *quantity* of key mitochondrial components are consistent with the small amount of published data on skeletal muscle from prior studies of long-term effects of anthracyclines. Yamada et al described an anthracycline-induced reduction in Complex I activity in rat diaphragm muscle^15^ while Lebrecht et al showed no change in gastrocnemius mtDNA-encoded COX1 or nuclear-encoded COX4 protein levels^11^. Our data are also consistent with prior data showing that, in contrast to cardiac muscle, anthracycline-containing chemotherapy does not cause a reduction in mtDNA content or increased levels of mtDNA deletions in skeletal muscle^11^.

It is impossible to use experimental rodent models to mimic current therapy of childhood ALL with precision, due to both changes in clinical treatment regimes over time and important species-specific differences in patterns of normal growth, development and metabolism. One other study in mice focused on evolution of chemo-resistance in ALL, used a more complex regime containing doxorubicin, dexamethasone, asparaginase and vincristine to mimic ALL treatment^30^. However, in the current study we were interested in longer term effects of chemotherapy treatment on host muscle tissue and chose to use a simplified regime combining doxorubicin with a lower dose of dexamethasone per cycle. Prolonged continuous use of higher doses of corticosteroids is a well-known cause of proximal myopathy, but the long-term impact of corticosteroids on mitochondrial function after treatment has not been well studied. In one report muscle biopsies from patients who had been taking an oral corticosteroid for months or years for a variety of chronic medical conditions^19^, revealed that very long-term steroid treatment was associated with an isolated reduction in muscle mitochondrial complex I activity and increased oxidative nuclear and mtDNA damage in patients, compared with steroid-naïve diseased and healthy controls^19^. However, our experiments used four cycles of 5-day treatments with dexamethasone at three-week intervals (i.e. a total of 20 days of treatment spread over three months), rather than continuous daily use. It is noteworthy that there is no prior data on the expected impact that this limited, intermittent steroid treatment might have on skeletal muscle mitochondria many weeks or months later. Further studies are needed to determine whether the observed long-term effects on skeletal muscle mitochondria are due to either doxorubicin or dexamethasone alone or an additive or even synergistic toxic effect of the combination treatment. There is good reason to believe that both may contribute to the observed deficits in skeletal muscle but the nature of any interaction is not yet clear, and this information will obviously be important for future potential preventative measures.

One of the strengths of this study is that prior studies of the effects of chemotherapy on mitochondrial function in muscle have frequently used only a few selected surrogate indices. In contrast, we performed comprehensive mitochondrial functional testing and employed a technique using saponin-permeabilized muscle fibers that avoids some of the bias inherent in using mechanically isolated mitochondria^31^. Our results provide the most complete functional assessments of the effects of chemotherapy treatment on muscle mitochondria to date. Moreover, our results include a detailed analysis of potential causes of mitochondrial dysfunction. Rather than being spared of any long-term toxic effects of anthracycline-containing chemotherapy, our data show that the mitochondria from skeletal muscle of mice treated with four cycles of doxorubicin and dexamethasone have substantial and persistent functional impairment, even 3 months after treatment was completed. The magnitude of mitochondrial respiratory impairment observed here (a 36% reduction in intrinsic mitochondrial respiratory capacity), equals or exceeds the most severe respiratory impairments reported in the literature for any disease or condition using the saponin-permeabilized myofiber preparation^32^,^33^,^34^, underscoring the persistent negative impact of the chemotherapy treatment.

Undoubtedly the cumulative long-term effects of doxorubicin and dexamethasone treatment may include effects on other organs or tissues. It is possible that the observed impairment of muscle mitochondrial function is related to indirect mechanisms rather than direct muscle-specific drug toxicity. However, the profile of muscle mitochondrial dysfunction in treated mice is distinct and does not correspond to patterns described for other putative indirect mechanisms such as premature aging-related changes, reduced food intake or reduced contractile activity levels. Thus, in aging muscle there is an increased sensitivity to mPTP opening, modest uncoupling and increased mitochondrial-derived proapoptotic signaling^21^,^34^, with fibre-type dependent reduction in mitochondrial content^34^. Similarly, long-term calorie restriction leads unchanged or elevated mitochondrial respiratory capacity and reduced ROS emission^35^. Finally, muscle disuse induces wasting with reduced muscle mitochondrial biogenesis and content^36^,^37^,^38^, increased absolute ROS production and oxidative damage of cytosolic proteins^39^.

Given the strong evidence for the involvement of oxidative damage in anthracycline-induced cardiac muscle dysfunction and the increase in ROS production per unit of respiration in muscle from treated mice of the current study (Fig 2), we looked for evidence of accumulation of oxidative damage. However, levels of the lipid peroxidation product 4-HNE were unchanged in whole muscle homogenates (Fig 4), demonstrating that despite causing persistent long-term mitochondrial dysfunction, chemotherapy treatment did not induce a parallel, sustained increase in oxidative damage at the whole tissue level. However, we recognize that the lack of evidence for increased oxidative damage at the whole muscle level after chemotherapy treatment, does not rule out accumulation of oxidative damage targeting specific mitochondrial proteins that are more sensitive to oxidative stress e.g. aconitase^40^ and mitochondrial adenine nucleotide translocase^41^.

In healthy tissues, overall mitochondrial functional integrity is maintained by a quality control process involving regular degradation (mitophagy) and replacement^35^. Impaired mitophagy alone can drive deterioration in mitochondrial function by disrupting normal quality control mechanisms^42^ and consistent with this idea, we observed a marked reduction in levels of Parkin (Fig 4), a protein which binds to VDAC on the outer mitochondrial membrane and triggers mitophagy^26^. This finding points to an acquired impairment of mitochondrial turnover, and we propose that the persistent defect mitochondrial respiration observed in the late group after chemotherapy is primarily due to a reduction in the rate of removal of mitochondria through mitophagy. Indeed, impaired mitophagic signaling may be a common mechanism contributing to long-term persistence of diverse patterns of mitochondrial dysfunction in muscle as a similar reduction in mitophagic potential (i.e. the Parkin-to-VDAC ratio) has also been reported in ageing muscle^21^.

To conclude, repeated administration of a combination of dexamethasone and doxorubicin leads to a profound impairment of skeletal muscle mitochondrial respiratory capacity and an increase in ROS production per unit respiration that persists three months after treatment has been completed. Further studies are needed to elucidate the molecular mechanisms underpinning this novel form of mitochondrial dysfunction more fully. In addition, it will be important to verify that similar changes are observed in adult survivors of ALL and even in other cancer survivors who received similar anti-cancer treatments. Confirming the presence of persistent muscle mitochondrial dysfunction will provide the mechanistic groundwork to guide intervention studies aimed at preserving muscle function and improving the long-term health status of these cancer survivors.

## Methods

### Animals

Female C57BL/6 mice aged 9 weeks (Harlan Laboratories, QC, Canada) were housed (5 mice per cage, 12 hour light/dark cycle) with free access to food (Teklad Global, 18% Protein Rodent Diet, Teklad Diets, Madison, WI) and water. Sealed phials of sterile pre-diluted doxorubicin and dexamethasone were supplied in light-opaque packaging by Jewish General Hospital pharmacy. Treated mice were injected i.p. with doxorubicin (10 mg/kg) on day one, and s.c. with dexamethasone (2.5 mg/kg) on days 1–5 of each cycle. Control mice were injected i.p. with equivalent volumes of sterile 0.9% saline. Chemotherapy was administered approximately every 3 weeks for a maximum of 4 cycles and mice body weights were recorded. At the end of the experiment, mice were euthanized by rapid asphyxiation with CO2. Two experimental groups were formed comprising the ‘early’ group euthanized at four weeks after the second cycle of chemotherapy (5 treated and control mice), and the ‘late’ group, euthanized 12 weeks after the end of the fourth cycle (10 treated and control mice). The experimental protocol was approved by the Animal Care Committee of the Montreal Children's Hospital Research Institute, and all experiments were performed in accordance with the guidelines of that committee.

### Tissue collection

Lower limb muscles from each animal were rapidly dissected and immediately frozen in liquid nitrogen and stored at −80°C. The right gastrocnemius (Gas) was used for mitochondrial function assessments (detailed below) while the left Gas was used for fiber size assessments, COX and SDH staining, immunoblotting studies and measurement of mtDNA copy number. The quadriceps muscle was used for measurement of mRNA levels and plantaris was used for mtDNA mutation analysis.

### Fiber size and type assessments

Serial sections of left Gas, 10 μm thick, were cut on a cryostat at −18°C. A first serial section was immunolabeled for myosin heavy chains (MHCs) I, IIx and laminin while a second serial section was MHC IIa and IIb and laminin as previously described^21^.

### Mitochondrial function studies

The right Gas was weighted and the muscle was manipulated manually into small fiber bundles which were then permeabilized with a mild detergent (saponin) as extensively described in Ref. 21. Different regions of the Gas muscle have different muscle fiber type composition and the following protocol was used to minimize variance in results due to this factor. Mitochondrial respiration (performed on the red Gas), ROS production (performed on the red Gas) and permeability transition pore sensitivity to Ca^2+^ were assessed (performed on the white Gas) at 37°C as described in detail previously^21^. Details on the buffer compositions, substrate and inhibitor concentrations and addition sequence can be found in Ref. 21. After each measurement was completed, bundles were removed and placed in liquid N_2_ and stored at −80°C for citrate synthase (CS) activity measures. Mitochondrial respiration, ROS production and calcium retention capacity were normalized per mg wet muscle weight. All experiments were analyzed with a bespoke program developed using the Igor Pro software (Wavemetrics, OR, USA).

### Citrate synthase assay

Enzymatic activity levels of the mitochondrial enzyme citrate synthase (CS) were determined spectrophotometrically on permeabilized red Gas myofibers frozen immediately after respirometry and ROS production using previously described methods^34^,^43^.

### Cytochrome c oxidase (COX) and succinate dehydrogenase (SDH) histochemisty

The sequential COX/SDH assay^44^ was performed on 10 μm sections of the left Gas to assess the activity of complex IV and complex II of the mitochondrial inner membrane respectively. If mtDNA integrity is compromised there will be a low to absent precipitation of the COX reaction product (oxidised diaminobenzidine tetrahydrochloride). Counter staining with the SDH media will stain these COX-deficient fibers blue and enable their easy identification, as SDH is nuclear encoded and it's activity is therefore not compromised by mtDNA mutations or depletion. Sections were incubated for 35 mins with COX reaction media followed by 40 mins incubation with the SDH reaction media. All incubations were performed in a humidified chamber at 37°C^45^.

### Molecular investigations into mtDNA integrity

Long-range polymerase chain reaction (PCR) was performed to look for the presence of deletions in the mtDNA. Total DNA was isolated from the plantaris muscle using a Qiagen DNeasy Blood & Tissue Kit according to manufacturer's instructions and eluted in 100 μl of dH_2_O. Primers were used to generate a 10475 bp product (Forward, 5406–5424, Reverse 15899–15799). Reactions were performed in a 25 μl volume consisting of 4 μl 10× dNTP's, 2.5 μl 10× reaction buffer, and 16.25 μl of dH2O, and 0.25 μl of TaKaRa LA Taq^TM^ Hot Start Version (TaKaRa Bio). 1 μl of sample DNA was added and amplification performed on a GeneAmp® PCR System (Applied Biosystems) thermal cycler under the following cycling conditions: 95°C for 10 minutes, thirty cycles of: 94°C for 45 seconds (denaturing), 58°C for 45 seconds (annealing), and 72°C for 1 minute (extension), with a final extension of 72°C for 8 minutes. Amplified fragments were separated on a 0.7% agarose gel and visualised using SYBR Safe® DNA Gel Stain (Life Technologies^TM^) and a SynGene G:Box gel imager (Syngene).

To determine mtDNA copy number 10 μm sections of left Gas were mounted on Arcturus®Pen Membrans Glass Slides (Life Technologies^TM^) and groups of 20 fibers were randomly chosen to be laser captured using the ArcturusXT^TM^ LCM microdissection system and Arcturus®CapSure® Macro LCM Caps (Life Technologies^TM^). Extraction of DNA was performed using 20 μl of lysis medium (500 nM Tris-HCL, 1% Tween 20,dH_2_O and proteinase K (Ambion®). The cells were then incubated at 55°C for 2 hr, and then 95°C for 10 mins. This procedure was performed on two groups of muscle fibers from each mouse with the sections being at least 70 μm apart from each other. A standard curve method of real-time PCR (qPCR) was employed to determine absolute mtDNA copy number in these samples^46^. Primers were used to generate a ND1 template of 981 bp in a standard PCR reaction, which was then purified using the QIAquick Gel Extraction Kit (Qiagen). The ND1 template DNA sample was then serially diluted for construction of a standard curve. The standard curve and experiment samples were then simultaneously amplified in a qPCR reaction. The reaction mixture consisted of 1 μl of DNA, 0.2 μl of forward and reverse primer (200 μM stock), 10 μl of Power SYBR®Green Master Mix (Life Technologies^TM^), and 8.6 μl of dH_2_O giving a 20 μl reaction. Cycle conditions were 10 mins at 95°C, 40 cycles of 15 secs at 95°C and 60 secs at 60°C, followed by a melt curve analysis to confirm specificity of the amplified product. Fluorescence levels were acquired at the end of each cycle. Copy number of the test sample was determined by reference to the standard curve constructed from the ND1 template samples, and then normalised to total cross-sectional area of the fibers used for the original DNA extraction.

### RNA studies

Approximately 150–200 mg of fresh quadriceps muscle was homogenised in 4 mls TRIzol, followed by phase separation with chloroform and RNA purification from the aqueous phase using the RNeasy Midi Kit with on-column DNase digestion (QIAGEN). Total RNA was reverse transcribed using SuperscriptTM III First-Strand Synthesis SuperMix for qRT-PCR (Invitrogen). The relative mRNA levels were determined by real-time PCR for Peroxisome proliferator-activated receptor gamma coactivator 1-α (Pgc1α), and mitochondrial transcription factor A (Tfam) using the following Taqman® probes: Mm01208835_m1, Mm_00447485_m1 respectively. Mouse 18S (hs99999901_s1), Cyclophilin (Mm02342429_g1) and RPLPO (Mm01974474_gH) were used as reference genes. Individual PCR reactions were run in triplicate. Amplification efficiencies for each probe were empirically determined and relative quantification was calculated using the Pfaffl method adjusted to normalise for all three reference genes^47^.

### Immunoblotting methods

Approximately 30–40 mg of Gas muscle was homogenized in 10 vols of an extraction buffer composed of 50 mM Tris base, 150 mM NaCl, 1% Triton X-100, 0.5% sodium deoxycolate, 0.1% SDS, and 10 μl/ml of a protease inhibitor cocktail (P8340; Sigma, St. Louis, MO, USA). The homogenate was centrifuged at 15,000 g for 15 min at 4°C. Aliquots of supernatant were heated in Laemmli buffer for 5 min at 40°C for mitochondrial inner membrane (OXPHOS) proteins, or 95°C for other proteins. Equal amounts of protein (30 μg) were loaded onto either 8 or 12% gels, electrophoresed by SDS-PAGE, and then transferred to polyvinylidene difluoride membranes (Life Sciences, Piscataway, NJ, USA). Membranes were incubated for 1 h at room temperature in a blocking buffer composed of 5% (w/v) nonfat dried milk in Tris-buffered saline containing 0.1% Tween 20 (TBS-T) and probed overnight at 4°C with the following primary antibodies (mouse monoclonal, 1:250 dilution, Abcam, Cambridge, MA, USA unless specified otherwise): anti-NDUFB8 (complex I; Ab110242), anti-SDHB (complex II; Ab14714), Anti-UQCRC2 antibody (Complex III, ab14745), Anti-MTCO1 antibody (Complex IV, ab14705), Anti-ATP5A antibody (ATP synthase, ab14748), anti- PRK8 (Parkin, sc32282; Santa Cruz Biotechnology, Santa Cruz, CA, USA), anti-VDAC1 (Ab14734, 1:500; Abcam), a rabbit polyclonal anti-4-hydroxy-2-nonenal antibody (HNE) (1:500; Calbiochem, San Diego, CA) and a rabbit polyclonal anti-β-tubulin (Ab6046, 1:500; Abcam) diluted in blocking buffer. Membranes were washed 6 times for 5 min each in TBS-T and subsequently incubated with appropriated HRP-conjugated secondary antibodies (Ab6721, 1:5000; Ab6728, 1:5000; Abcam) diluted in blocking buffer 1 h at room temperature. Protein signals were detected using enhanced chemiluminescence substrate (24080; Thermo Scientific; Waltham, MA, USA), imaged with a G-Box chem imaging system (Syngene, Cambridge, UK) and analyzed using ImageJ (U.S. National Institutes of Health, Bethesda, MD, USA).

### Statistical analysis

Unless otherwise mentioned in figure legends, comparisons comparison between control and treated mice were performed using unpaired two-tailed Student's t tests. Values of P < 0.05 were considered significant.

## Author Contributions

R.T.J., C.S.B., B.M., J.R. and R.T.H. designed the study; C.S.B. and B.M. performed the live animal experimentation; C.S.B., S.S., F.P.S., G.G., H.M. and E.A.L. collected tissues; G.G. and N.S. performed the mitochondrial function studies; C.S.B., E.A.L., H.M. and R.T.J. mRNA expression; S.S., Y.K. and T.T. DNA mutation analysis; F.P.S., N.S. and R.T.H. muscle fibre typing and morphometry; M.V., G.G. and S.C. protein expression. R.T.J., G.G., C.S.B. and R.T.H. wrote the manuscript. G.G. and R.T.J. prepared figures. All authors reviewed the manuscript.
